# Comment on “Osteopontin, Macrophage Migration Inhibitory Factor and Anti-Interleukin-8 Autoantibodies Complement CA125 for Detection of Early Stage Ovarian Cancer” *Cancers* 2019, *11*, 596: Markers for Early Detection of Ovarian Cancer

**DOI:** 10.3390/cancers11091307

**Published:** 2019-09-05

**Authors:** Gil Mor, Thomas Rutherford, Ayesha Alvero

**Affiliations:** 1 C.S Mott Center for Human Development, Department of Obstetrics and Gynecology, Wayne State University, Detroit, MI 48201, USA; 2 Department of Obstetrics, Gynecology & Reproductive Science, Yale University New Haven, CT 06520, USA; 3 Department of Obstetrics and Gynecology, University of South Florida, Tampa, FL 33620, USA

We read with interest the recent publication by Guo et al. [1] where they describe the identification and characterization of Osteopontin (OPN), Macrophage Migration Inhibitory (MIF), and anti-interleukin-8 autoantibodies as markers for early detection of ovarian cancer. The authors identified these proteins from a panel of 10 ovarian cancer associated protein antigens and 12 autoantibodies reactive with cancer associated proteins. 

The authors evaluated the specificity of each protein to detect early or late stage of ovarian cancer as well as in combination with CA125. They found that the combination CA125, OPN and MIF had the highest AUC = 0.999 (95% CI:0.998–1.000). They also applied different models consisting of different combinations of the identified markers. 

Although the study is interesting and well-designed, it is not novel. The study only confirms already published data, although they ignored those publications. 

Our group reported in a first publication in PNAS [2], in 2005, the identification of OPN and MIF out of 169 proteins. The analytes were then validated using additional 106 healthy females and 100 patients with Epithelial Ovarian Cancer (EOC) (24 stage I_II and 76 stage III_IV). Upon sample decoding, the results were analyzed by using three different classification algorithms and a binary code methodology. The four-analyte test was further validated in a blind binary code study by using 40 additional serum samples from normal and EOC cancer patients. No single protein could completely distinguish the cancer group from the healthy controls. However, the combination of the analytes exhibited a better sensitivity and specificity. A similar experimental approach was used by Guo et al. but they did not refer to the 2005 report.

In a second report published in Clinical Cancer Research in 2008 [3], we reported that the combination of these markers with CA125 provides a higher sensitivity and specificity for the detection of early and late cases. We analyzed 362 healthy controls and 156 newly diagnosed ovarian cancer patients. All markers were evaluated in a training set (181 samples from the control group and 113 samples from ovarian cancer patients) and a test set (181 sample control group and 43 ovarian cancer).

In the study we also described multiple combination models, similar to those described in the Gao et al. report and concluded that the combination of the markers gave the best sensitivity and specificity, with OPN and MIF as the top markers. In spite of the similarities of the approach and outcome of the studies, Guo et al. ignored these two reports in their manuscript and present their approach and findings as novel.

We are pleased that the Guo study confirm our published findings, however is incorrect to ignore published data with claims of originality. 
